# Mimicking descending necrotizing mediastinitis as acute myocardial infarction in a patient with severe coronary artery disease: A case report

**DOI:** 10.1097/MD.0000000000036571

**Published:** 2023-12-08

**Authors:** Yu Jung Jung, Jong-Il Park

**Affiliations:** a Division of Cardiology, Yeungnam University Medical Center, Daegu, Republic of Korea.

**Keywords:** acute myocardial infarction, descending necrotizing mediastinitis, ppercutaneous coronary intervention

## Abstract

**Rationale:**

It is a crucial disease that descending necrotizing mediastinitis need to be treated promptly with proper antibiotics and drainage. The characteristics of its symptoms such as chest pain are difficult to distinguish from acute myocardial infarction.

**Patient concerns:**

An 80-year-old female presented with severe squeezing chest pain. The cardiac marker was elevated. And coronary angiography showed the significant coronary stenosis. Although the revascularization through percutaneous coronary intervention was completed successfully, the patient still presented chest pain. Computed tomography of neck revealed that hypodense heterogeneous lesions with clear and distinguishable margin extended from the deep neck to mediastinum diffusely.

**Diagnoses:**

The patient was diagnosed with descending necrotizing mediastinitis.

**Interventions:**

Percutaneous catheter insertion to patient’s abscess lesion at was performed.

**Outcomes:**

Catheter drainage of descending necrotizing mediastinitis led to an improvement in the patient’s condition.

**Lesson:**

Descending necrotizing mediastinitis made chest paint with elevated cardiac enzyme mimicked myocardial infarction.

## 1. Introduction

It is known that the descending necrotizing mediastinitis (DNM) is a potentially lethal disease of the mediastinum with a mortality rate as high as 40%.^[1]^ Differential diagnosis for chest pain is often complicated by the presence of DNM, leading to potential misdiagnosis as acute myocardial infarction (AMI).^[2–4]^ In this case, the patient presented with chest pain and an elevation of troponin I (TnI), which raised suspicions of AMI. Additionally, the patient’s medical history, laboratory results, the presence of a severe coronary stenosis observed on coronary angiography. Here, we present a rare case that the diagnosis of chest pain was challenging due to the presence of severe coronary artery stenosis leading to the initial diagnosis of AMI, accompanied by concurrent DNM.

## 2. Case report

An 80-year-old female presented to the emergency room with chest pain. She experienced recurring episodes of cramping chest pain episodes lasting for approximately 3 to 4 minutes prior to seeking medical attention. The patient reported chest discomfort in the middle of her chest and difficulty breathing, both at rest and during physical exertion. Given her medical history, which included atrial fibrillation, type 2 diabetes mellitus, hyperthyroidism, and chronic kidney disease. Initial vital sign was stable. Blood pressure was 140/90 mm Hg, heart rate 75 beats per minute, respiratory rate was 16 breaths per minute, and body temperature was 36.5ºC.

**Figure 1.**
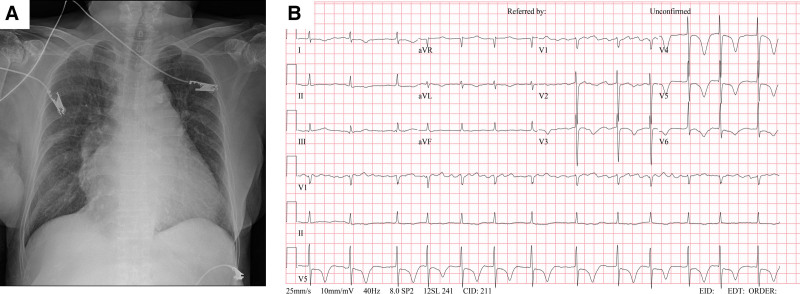
(A) Chest X-ray and (B) electrocardiogram.

The chest X-ray revealed cardiomegaly with intact cardiac margin (Fig. 1A). Additionally, the level of TnI was elevated at 0.482 ng/mL, indicating cardiac muscle damage. Furthermore, the level of pro-brain natriuretic peptide exceeded the maximum value of 35,000 pg/mL as determined by the measuring machine. The patient’s electrocardiogram (ECG) exhibited an irregular heart rate and rhythm, consistent with atrial fibrillation, with evidence of strained T-waves observed over the precordial leads (Fig. 1B).

**Figure 2.**
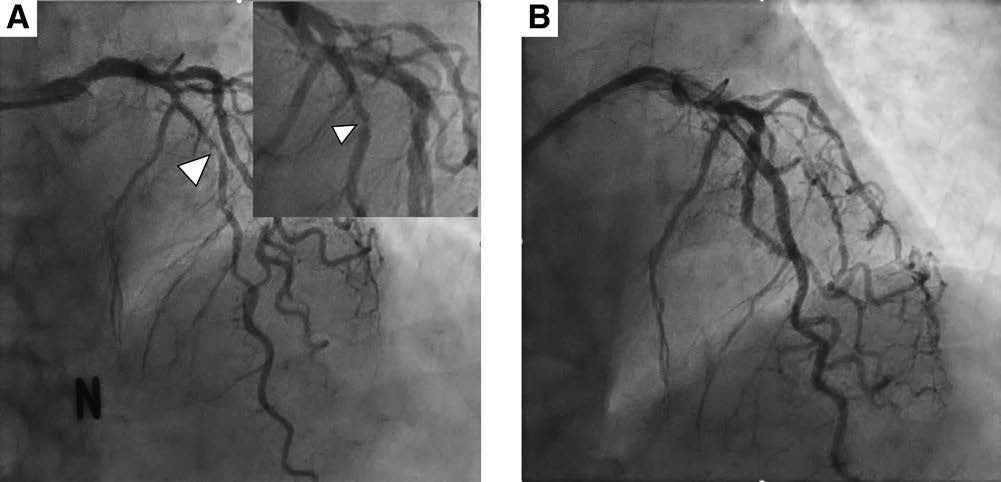
Coronary angiography. (A) Before stent insertion at the lesion of stenosis on left anterodescending artery (arrowhead). (B) After percutaneous coronary intervention.

**Figure 3.**
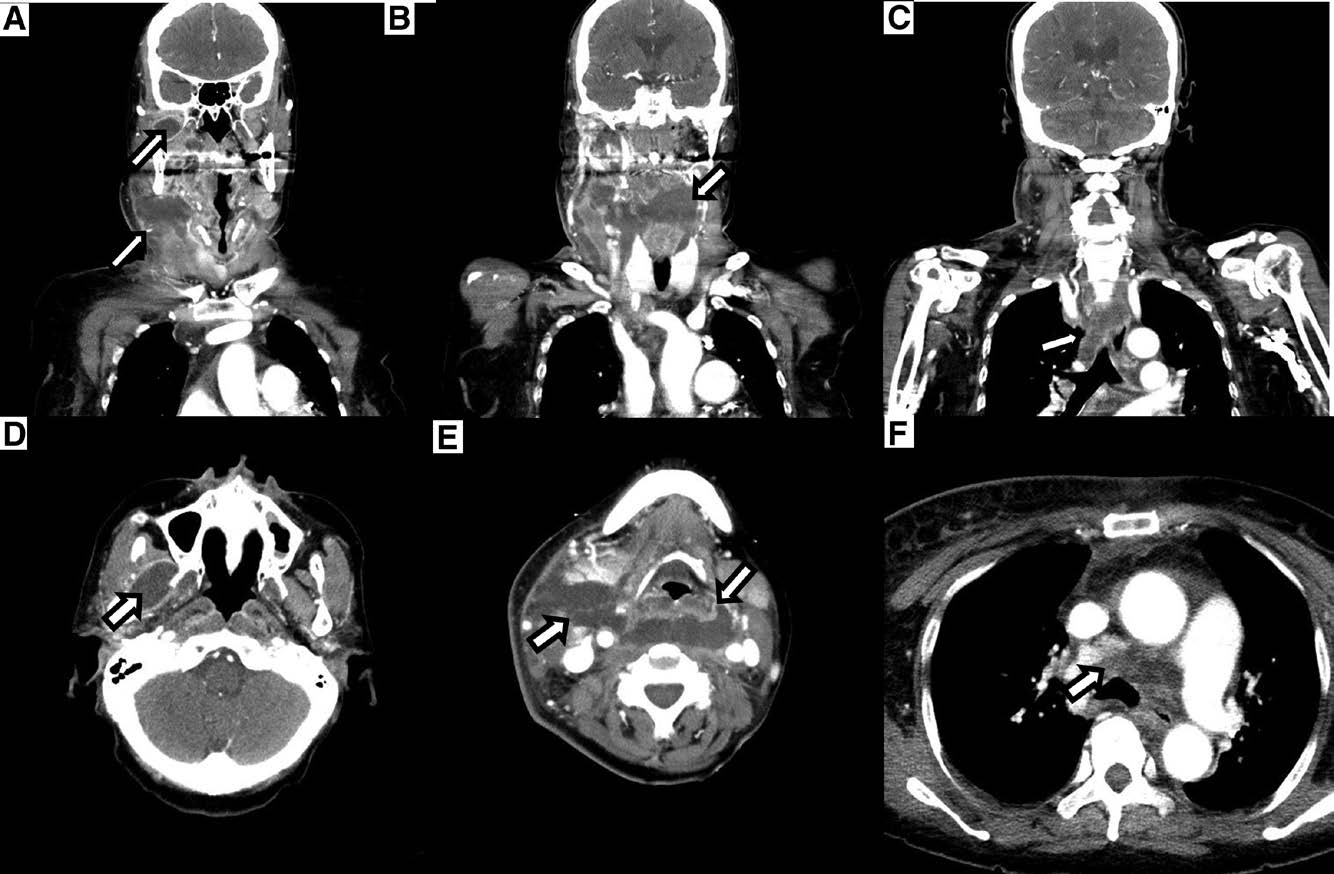
Neck CT with intravenous contrast. (A–C) Coronal view of CT show abscess formation from the right maxillary sinus to mediastinal space. (D–F) Horizontal view of CT shows abscess formation from right maxillary sinus to mediastinal space as same as coronal view. CT = computed tomography.

**Figure 4.**
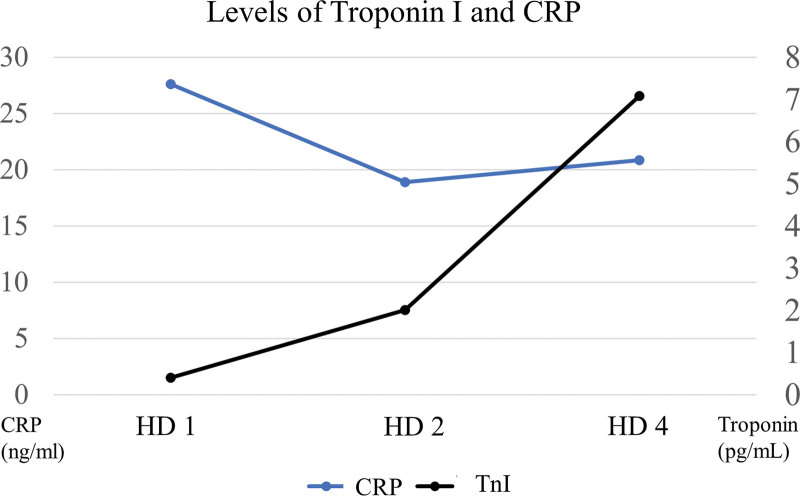
Level of TnI and CRP. After percutaneous coronary intervention, level of troponin increased. CRP = C-reactive protein, TnI = troponin I.

Due to ongoing chest pain and elevated troponin-I level, we promptly considered to check coronary artery. Coronary angiography showed significant stenosis from the proximal to mid left anterior descending artery. We performed percutaneous coronary intervention (PCI) (Fig. 2). After successful PCI, the patient continued to complain chest discomfort and breathing difficulty even at rest. A neck computed tomography (CT) with intravenous contrast was conducted. Revealing an abscess formation in retropharyngeal space extending from the maxillary sinus of the right sphenoidal bone to mediastinal spaces (Fig. 3). This abscess caused chest pain and difficulty breathing as well as compression of airways from the outside of trachea. Subsequently, a thorough review of the patient’s medical history was conducted, revealing that she had undergone wisdom teeth extraction 1 week prior. The patient’s condition rapidly getting worsened, necessitating the use of mechanical ventilation to provide adequate oxygen support. Furthermore, compromised airway due to tracheal narrowing and collapse caused by the abscess required immediate intervention. A percutaneous catheter was inserted under ultrasound guidance to facilitate drainage of the abscess. Additionally, a change in intravenous antibiotics was implemented, transitioning from ceftriaxone with azithromycin to carbapenem with glycopeptide. After the administration of broad-spectrum antibiotics and the drainage of the abscess through the percutaneous catheter, the symptom of the patients was completely recovered. And there was a decline in C-reactive protein levels from 27 to 18 mg/dL, indicating a reduction in inflammation (Fig. 4). In the echocardiography, although the left ventricular systolic function was reserved, the apex of right ventricle showed regional wall motional abnormality with decreased right ventricle systolic function. The troponin-I level showed a persistent rise from 0.4 to 7.09 ng/mL, like the findings of echocardiogram (Fig. 4).

## 3. Discussion

The Disease originating from Deep Neck Infection (DNM) is a potentially lethal mediastinal condition, with a mortality rate as high as 40%.^[1]^ The primary site commonly originates from the pharynx or oral cavity, specifically odontogenic infections from the second and third molars.^[5]^ Due to factors such as gravity, respiration, and negative intrathoracic pressure, the infection can progress downward into the mediastinal space.^[6]^ When it spreads quickly down to the mediastinal space, it make chest pain.^[7]^ Diagnostic criteria for DNM were established by Estrera et al^[8]^ in 1983: clinical manifestations of severe infection, demonstration of characteristic radiographic findings, documentation of necrotizing mediastinal infection in operation, and establishment of oropharyngeal/cervical infection with DNM relationship. CT is performed to diagnosis of DNM and identify the location of lesion.^[9,10]^ Effective management of DNM involves the use of appropriate antibiotics, as well as the essential procedures of debridement and drainage.^[9–11]^

Ischemic type chest discomfort, dyspnea, nausea, unexplained weakness, or a combination of these symptoms imply AMI.^[12]^ Upon the commencement of clinical history-taking, an ECG and serum troponin test are performed to diagnose the condition when a patient presenting at the emergency room complains an acute chest pain.^[13,14]^ Patient with myocardial infarction has to be admitted to hospital and subsequently receive guideline-directed medical treatment along with invasive coronary angiography.^[15,16]^ If a patient experiences persistent chest pain with changed ECG rhythm and elevation of cardiac marker, which indicates AMI.^[12,13,15,17]^ Approximately 10% of patients with chest pain receive a diagnosis of acute coronary syndrome.^[18]^ It’s important to note that chest pain can arise from not only cardiac causes but also respiratory and musculoskeletal origins.^[14]^

In our case, the symptoms and signs appeared at the patient’s arrival of ER indicated the acute coronary syndrome. Without doubt, we got into the catheter lab to revascularize if it is demanded. The patient still experienced persistent chest discomfort after a successful PCI procedure, and there was an increase in the patient’s serum troponin levels. This necessitated considering a range of potential diagnoses for the chest discomfort. Given the circumstances, it was essential to investigate other potential causes of chest pain.^[14]^ This investigation involved performing CT, collecting more blood samples, and conducting a thorough medical history review. Reviewing the history taking of patient, it was the extraction of wisdom teeth 2 weeks before the patient’s chest discomfort. Subsequently, it was determined that an infection, specifically DNM, was causing the patient’s chest pain and mimicking the symptoms of myocardial infarction. Without delay, appropriate measures were taken, including the drainage of the DNM and the administration of suitable intravenous antibiotics.

Especially in our case, the diagnosis of DNM posed an even greater challenge due to the presence of coronary artery stenosis lesions observed during coronary angiography, along with the patient experiencing chest pain and an elevation of troponin levels. Despite the perplexing nature of this situation, we were able to identify the presence of DNM in a timely manner. As a result, both the coronary lesion and the DNM were effectively treated.

## 4. Conclusion

This study highlights the potential for DNM to be misdiagnosed as AMI, particularly when considering the alterations of cardiac enzyme and elevated level of TnI. Moreover, the presence of a severe stenosis on coronary angiography may further delay the correct diagnosis. Therefore, a comprehensive assessment of the patient’s medical history is of utmost important to ensure an accurate and timely diagnosis.

## Acknowledgements

This case report was written according to the CARE guidelines.

## Author contributions

**Conceptualization:** Yu Jung Jung, Jong-Il Park.

**Supervision:** Jong-Il Park.

**Validation:** Jong-Il Park.

**Writing – original draft:** Yu Jung Jung.

**Writing – review & editing:** Yu Jung Jung, Jong-Il Park.

## References

[1] Ho CY Chin SC Chen SL. Management of descending necrotizing mediastinitis, a severe complication of deep neck infection, based on multidisciplinary approaches and departmental co-ordination. Ear Nose Throat J. 2022:1455613211068575. 35023759 10.1177/01455613211068575

[2] Catarino PA Westaby S. Postcardiac surgery mediastinitis mimicking acute inferior myocardial infarction. J Card Surg. 2000;15:309–12. 11599821 10.1111/j.1540-8191.2000.tb00462.x

[3] Kataoka E Kimura M Iwai T. ST-segment elevation mimicking inferior wall myocardial infarction caused by right tension pneumothorax. Circ J. 2022;86:1590. 35545530 10.1253/circj.CJ-22-0135

[4] Jung HW. ST-segment elevation due to myocardial invasion of lung cancer mimicking ST elevation myocardial infarction: a case report. Medicine (Baltimore). 2021;100:e26088. 34011132 10.1097/MD.0000000000026088 PMC8137083

[5] Parhiscar A Har-El G. Deep neck abscess: a retrospective review of 210 cases. Ann Otol Rhinol Laryngol. 2001;110:1051–4. 11713917 10.1177/000348940111001111

[6] Kanafani ZA Arduino JM Muhlbaier LH. Incidence of and preoperative risk factors for Staphylococcus aureus bacteremia and chest wound infection after cardiac surgery. Infect Control Hosp Epidemiol. 2009;30:242–8. 19199534 10.1086/596015

[7] Pravita S Wibisono S Dewi IP. Prompt and aggressive treatment of deep neck infection in neglected diabetic patient: a case report and literature review. Caspian J Intern Med. 2023;14:406–11. 37223280 10.22088/cjim.14.2.406 PMC10201117

[8] Estrera AS Landay MJ Grisham JM. Descending necrotizing mediastinitis. Surg Gynecol Obstet. 1983;157:545–52. 6648776

[9] Kocher GJ Hoksch B Caversaccio M. Diffuse descending necrotizing mediastinitis: surgical therapy and outcome in a single-centre series. Eur J Cardiothorac Surg. 2012;42:e66–72. 22761501 10.1093/ejcts/ezs385

[10] Misthos P Katsaragakis S Kakaris S. Descending necrotizing anterior mediastinitis: analysis of survival and surgical treatment modalities. J Oral Maxillofac Surg. 2007;65:635–9. 17368356 10.1016/j.joms.2006.06.287

[11] Reuter TC Korell V Pfeiffer J. Descending necrotizing mediastinitis: etiopathogenesis, diagnosis, treatment and long-term consequences – a retrospective follow-up study. Eur Arch Otorhinolaryngol. 2023;280:1983–90. 36478116 10.1007/s00405-022-07769-x PMC9988808

[12] Anderson JL Morrow DA. Acute myocardial infarction. N Engl J Med. 2017;376:2053–64. 28538121 10.1056/NEJMra1606915

[13] Lee TH Goldman L. Evaluation of the patient with acute chest pain. N Engl J Med. 2000;342:1187–95. 10770985 10.1056/NEJM200004203421607

[14] Gulati M Levy P Mukherjee D. 2021 AHA/ACC/ASE/ASNC/CHEST/SAEM/SCCT/SCMR guideline for the evaluation and diagnosis of chest pain. J Am Coll Cardiol. 2021;144:e368–e454.

[15] Amsterdam EA Wenger NK Brindis RG. 2014 AHA/ACC guideline for the management of patients with non-ST-elevation acute coronary syndromes: a report of the American College of Cardiology/American Heart Association Task Force on Practice Guidelines. J Am Coll Cardiol. 2014;64:e139–228. 25260718 10.1016/j.jacc.2014.09.017

[16] Gulati M Levy PD Mukherjee D. 2021 AHA/ACC/ASE/CHEST/SAEM/SCCT/SCMR guideline for the evaluation and diagnosis of chest pain: executive summary: a report of the American College of Cardiology/American Heart Association Joint Committee on Clinical Practice Guidelines. Circulation. 2021;144:e368–454. 10.1161/CIR.0000000000001030 34709928

[17] Reichlin T Hochholzer W Bassetti S. Early diagnosis of myocardial infarction with sensitive cardiac troponin assays. N Engl J Med. 2009;361:858–67. 19710484 10.1056/NEJMoa0900428

[18] Fanaroff AC Rymer JA Goldstein SA. Does this patient with chest pain have acute coronary syndrome?: the rational clinical examination systematic review. JAMA. 2015;314:1955–65. 26547467 10.1001/jama.2015.12735

